# Retrospective natural history of thymidine kinase 2 deficiency

**DOI:** 10.1136/jmedgenet-2017-105012

**Published:** 2018-03-30

**Authors:** Caterina Garone, Robert W Taylor, Andrés Nascimento, Joanna Poulton, Carl Fratter, Cristina Domínguez-González, Julie C Evans, Mariana Loos, Pirjo Isohanni, Anu Suomalainen, Dipak Ram, M Imelda Hughes, Robert McFarland, Emanuele Barca, Carlos Lopez Gomez, Sandeep Jayawant, Neil D Thomas, Adnan Y Manzur, Karin Kleinsteuber, Miguel A Martin, Timothy Kerr, Grainne S Gorman, Ewen W Sommerville, Patrick F Chinnery, Monika Hofer, Christoph Karch, Jeffrey Ralph, Yolanda Cámara, Marcos Madruga-Garrido, Jana Domínguez-Carral, Carlos Ortez, Sonia Emperador, Julio Montoya, Anupam Chakrapani, Joshua F Kriger, Robert Schoenaker, Bruce Levin, John L P Thompson, Yuelin Long, Shamima Rahman, Maria Alice Donati, Salvatore DiMauro, Michio Hirano

**Affiliations:** 1 Department of Neurology, Columbia University Medical Center, New York City, New York, USA; 2 MRC Mitochondrial Biology Unit, Cambridge Biomedical Campus, Cambridge, UK; 3 Wellcome Trust Centre for Mitochondrial Research, Institute of Neuroscience, The Medical School, Newcastle University, Newcastle upon Tyne, UK; 4 Neuromuscular Unit, Hospital Universitario 12 de Octubre, Madrid, Spain; 5 Nuffield Department of Obstetrics and Gynaecology, University of Oxford, Oxford, UK; 6 Oxford Medical Genetics Laboratories, Oxford University Hospitals NHS Foundation Trust, Oxford, UK; 7 Centre for Biomedical Network Research on Rare Diseases (CIBERER), Instituto de Salud Carlos III, Madrid, Spain; 8 Instituto de Investigación, Hospital Universitario 12 de Octubre, Madrid, Spain; 9 Neurology Department, Hospital de Pediatría ‘Prof. Dr JP Garrahan’, Buenos Aires, Argentina; 10 Research Programs Unit, Molecular Neurology, Biomedicum Helsinki, University of Helsinki, Helsinki, Finland; 11 Department of Child Neurology, Children’s Hospital, University of Helsinki, Helsinki University Hospital, Helsinki, Finland; 12 Neuroscience Center, University of Helsinki, Helsinki, Finland; 13 Department of Neurology, Helsinki University Hospital, Helsinki, Finland; 14 Department of Paediatric Neurology, Royal Manchester Children’s Hospital, Manchester, UK; 15 UOC Neurology and Neuromuscular Diseases, Department of Clinical and Experimental Medicine, University of Messina, Messina, Italy; 16 Paediatric Neurology, Oxford University Hospitals NHS Foundation Trust, Oxford, UK; 17 Paediatric Neurology, University Hospital Southampton NHS Foundation Trust, Southampton, UK; 18 Dubowitz Neuromuscular Centre, Great Ormond Street Hospital for Children NHS Foundation Trust, London, UK; 19 Pediatric Neurology, Faculty of Medicine, Universidad de Chile, Clínica Las Condes, Santiago, Chile; 20 Paediatric Neurology, St George’s University Hospitals NHS Foundation Trust, London, UK; 21 Wellcome Trust Centre for Mitochondrial Research, Institute of Genetic Medicine, Newcastle University, Newcastle upon Tyne, UK; 22 Department of Neuropathology, Oxford University Hospitals NHS Foundation Trust, Oxford, UK; 23 Department of Neurology, University of California San Francisco, San Francisco, California, USA; 24 Research Group on Neuromuscular and Mitochondrial Disorders, Vall d’Hebron Institut de Recerca, Barcelona, Spain; 25 Centre for Biomedical Network Research on Rare Diseases (CIBERER), Instituto de Salud Carlos III, Barcelona, Spain; 26 Sección de Neuropediatría, Hospital Universitario Virgen del Rocío, Instituto de Biomedicina de Sevilla, Seville, Spain; 27 Neuromuscular Unit, Department of Neurology, Hospital Sant Joan de Déu, CIBERER, ISCIII, Universitat de Barcelona, Barcelona, Spain; 28 Department of Biochemistry and Molecular Biology, University of Zaragoza–CIBERER–Instituto de investigaciones Sanitarias de Aragón, Zaragoza, Spain; 29 Metabolic Unit, Great Ormond Street Hospital NHS Foundation Trust, London, UK; 30 Department of Biostatistics, Mailman School of Public Health, Columbia University Medical Center, New York City, New York, USA; 31 Mitochondrial Research Group, Genetics and Genomic Medicine, UCL Great Ormond Street Institute of Child Health, London, UK; 32 Metabolic and Neuromuscular Unit, Meyer Hospital, Florence, Italy

**Keywords:** metabolic disorders, muscle disease, neuromuscular disease, clinical genetics

## Abstract

**Background:**

Thymine kinase 2 (TK2) is a mitochondrial matrix protein encoded in nuclear DNA and phosphorylates the pyrimidine nucleosides: thymidine and deoxycytidine. Autosomal recessive *TK2* mutations cause a spectrum of disease from infantile onset to adult onset manifesting primarily as myopathy.

**Objective:**

To perform a retrospective natural history study of a large cohort of patients with TK2 deficiency.

**Methods:**

The study was conducted by 42 investigators across 31 academic medical centres.

**Results:**

We identified 92 patients with genetically confirmed diagnoses of TK2 deficiency: 67 from literature review and 25 unreported cases. Based on clinical and molecular genetics findings, we recognised three phenotypes with divergent survival: (1) infantile-onset myopathy (42.4%) with severe mitochondrial DNA (mtDNA) depletion, frequent neurological involvement and rapid progression to early mortality (median post-onset survival (POS) 1.00, CI 0.58 to 2.33 years); (2) childhood-onset myopathy (40.2%) with mtDNA depletion, moderate-to-severe progression of generalised weakness and median POS at least 13 years; and (3) late-onset myopathy (17.4%) with mild limb weakness at onset and slow progression to respiratory insufficiency with median POS of 23 years. Ophthalmoparesis and facial weakness are frequent in adults. Muscle biopsies show multiple mtDNA deletions often with mtDNA depletion.

**Conclusions:**

In TK2 deficiency, age at onset, rate of weakness progression and POS are important variables that define three clinical subtypes. Nervous system involvement often complicates the clinical course of the infantile-onset form while extraocular muscle and facial involvement are characteristic of the late-onset form. Our observations provide essential information for planning future clinical trials in this disorder.

## Introduction

Thymidine kinase 2 (TK2) is a nuclear-encoded mitochondrial enzyme that catalyses the conversion of deoxycytidine and thymidine nucleosides to their nucleoside monophosphates that, in turn, are further phosphorylated to generate deoxynucleoside triphosphates that are incorporated into replicating mitochondrial DNA (mtDNA). Autosomal recessive deficiency of TK2 was initially described in 2001 by Saada and colleagues^1^ in four children with severe myopathy, elevated creatine kinase, multiple defects of mitochondrial respiratory chain activity and low mtDNA copy number in muscle tissue. Since the original description of TK2 deficiency, 63 additional patients have been described in isolated case reports and small series with heterogeneous clinical and molecular characteristics indicating that TK2 deficiency encompasses a disease spectrum.^2–25^ The lack of a comprehensive study depicting the natural history and defining the clinical, molecular and biochemical features has limited early recognition of the disease, prognostic information for patients and families, and planning for potential future clinical trials.

## Materials and methods

This multicentre retrospective study focuses on patients with TK2 deficiency. Informed consent for anonymous publication of the patients’ clinical features and analyses of DNA samples, skin-derived fibroblast cell lines and muscle tissues were obtained from all study participants under a Columbia University Medical Centre Institutional Review Board–approved protocol or local ethics committee approval of the referring clinical centre. Systematic literature review using the terms ‘Thymidine kinase 2 deficiency’, ‘Thymidine kinase 2’ and ‘TK2’ from October 2001 to January 2016 was conducted in PubMed. Physicians caring for reported patients were invited to provide follow-up data and to contribute additional unreported cases with TK2 deficiency confirmed by molecular genetic testing. Mitochondrial disease research centres in Argentina (one), Chile (one), Finland (one), Italy (one), Spain (two), UK (three) and the USA (one) participated in the study. We collected the following information: age at onset, sign/symptoms, motor performance (maximal motor ability, age when wheelchair-bound, as well as severity and distribution of weakness), respiratory involvement (use of non-invasive ventilation and invasive ventilation), cardiomyopathy, bulbar involvement (gastrostomy, solid or liquid dysphagia, and facial weakness), central and peripheral nervous system involvement (cognitive impairment, brain MRI abnormalities, encephalopathy, seizures, motor neuron involvement and peripheral neuropathy), non-neurological signs/symptoms indicative of multisystemic involvement (kidney, heart, bone and liver abnormalities), survival, cause of death, serum creatine kinase (CK) levels, electromyography (EMG) studies and muscle histology and biochemistry.

Molecular genetic studies were performed in reference centres as previously described^25–27^: direct Sanger sequencing of *TK2* (NM_004614.4) or next-generation whole-exome or mitochondrial nuclear gene panel sequencing for genetic diagnosis, long PCR or Southern blot analysis to detect multiple mtDNA deletions and quantitative PCR for mtDNA copy number in muscle tissue. TK2 activity was assessed in cultured fibroblasts of patients.

All statistical analysis was performed using SAS V.9.4. Clinical subgroup comparisons used 1 or 2 df χ^2^ tests and survival analyses employed log-rank or Wilcoxon tests, as appropriate.

## Results

### Clinical features

We identified and obtained natural history data for 92 patients (55 men, 37 women) with genetically confirmed diagnoses of TK2 deficiency (online supplementary tables 1–3). For 67 of these, who had been reported, data were obtained from literature review. For 25, previously unreported, data were provided by study contributors.

10.1136/jmedgenet-2017-105012.supp1 Supplementary data

Age at disease onset ranged from birth to 72 years of age. We stratified patients into three groups based on onset age. Among the 90 for whom this was available, 39 (43.3%) had infantile onset (≤1 year), 37 (41.1%) childhood onset (>1 and <12 years) and 14 (15.6%) late onset (>12 years). In all but one of the late-onset group, initial manifestations presented after adolescence (>17 years). The proportions of male and female patients in the three groups did not differ significantly (P=0.46, 2 df χ^2^ test).

All patients had proximal muscle weakness as the first sign of disease or during the clinical course. CK levels, available in 57 out of 92 patients, were elevated in all from 1.3-fold to 30-fold above the upper limit of normal (range 272–6500 U/L). In five patients, CK levels were reportedly increased, but values were not available. In seven patients (two infantile-onset, four childhood-onset and one late-onset disease), CK values were transiently in the normal range. CK elevation was associated with myoglobinuria (one) and rhabdomyolysis (two). Nerve conduction studies (NCSs) and EMG were performed in 42 patients, and results classified as myopathic changes defined by polyphasic short-duration low-amplitude motor unit potentials (MUPs) in 33/42 (78.6%), ‘myopathic and neuropathic’ changes in 3/42 (7.2%), sensory axonal neuropathy by NCS in one (2.4%), chronic denervation by EMG in another patient (2.4%), low-amplitude MUPs in a facial muscle in one patient (2.4%), isolated ‘neuropathic’ changes in 3/42 (7.1%), sensory axonal peripheral neuropathy in one (2.4%) and no abnormalities in three patients (7.1%). Although neurogenic abnormalities were detected in 6/34 (17.6%) electrophysiological studies, only two patients had sensory peripheral neuropathy.

Global motor function was severely impaired in 50/61 (82% with Karnofsky or Lansky Performance Status <50) who were wheelchair-bound at the last follow-up. Eleven patients (three children, nine adults; 18%) had motor function compatible with nearly normal daily life at last follow-up; they were able to walk short distances independently, climb stairs or both. Data from motor rating scales were not available for 31 patients. Muscle weakness was most prominent in the first year of life in the infantile-onset form, with 26 of 32 (81%) never acquiring the ability to walk independently and virtually all (30/32; 94%) non-ambulatory by age 4 years, with only one still walking at age 4 years and another as an adult^3^ (data were not available in seven patients). In contrast, 19/30 (63%) of childhood-onset patients became wheelchair-bound within 10 years of disease onset except for two patients; one was ambulatory until age 15 (11 years after onset) and the other walked until age 22 (20 years after onset).^13^ Of the ambulatory childhood-onset patients, the majority (8/11, 64%) were within 10 years of disease onset, but four were walking more than 10 years after onset at ages 15 (11 years post-onset), 25 (23.5 years post-onset), 28 (19 years post-onset) and 29 years (27.9 years post-onset) (data were not available in seven patients). In contrast to the infantile-onset/childhood-onset patients, late-onset cases never became wheelchair-bound (data were not available in eight patients).

Respiratory muscles were severely compromised in 33/51 (65%) patients, who required mechanical ventilation or nocturnal/continuous non-invasive ventilation (data were not available in 41 patients). The vast majority of infantile-onset patients required ventilation (17/19, 89.5%) while ventilatory support was used in about half the childhood-onset (12/22, 54.4%) and late-onset (4/9, 44.4%) patients.

**Figure 1:**
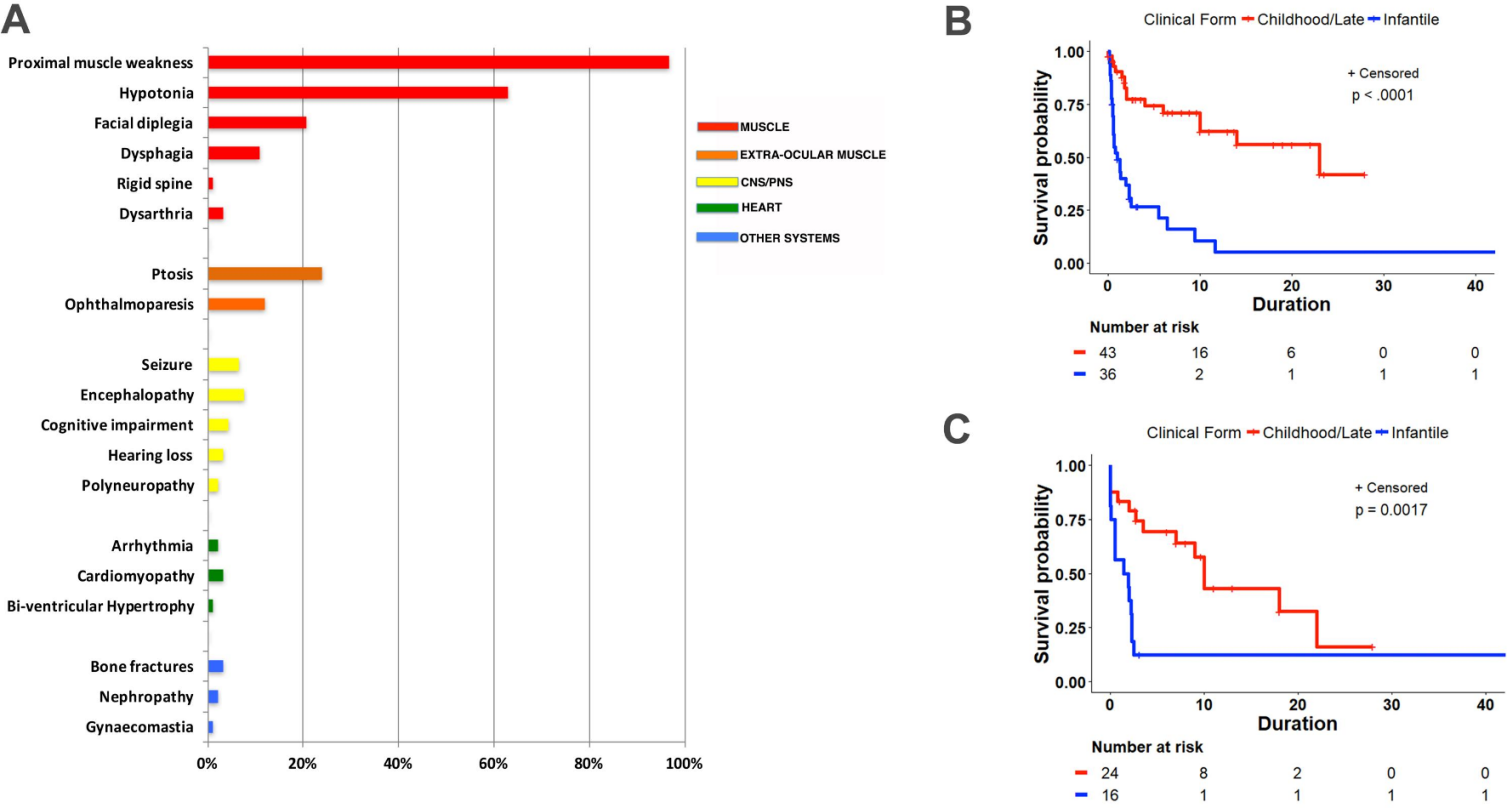
Manifestations and survival curves of thymine kinase 2–deficient patients. (A) Bar graph showing the prevalence of symptoms in different organs/tissues in the cohort of patients. (B) Post-onset survival in infantile-onset and childhood-onset/late-onset patients (n=79). (C) Event-free survival (time from onset to ventilation or death) in infantile-onset and childhood-onset/late-onset patients (n=40). CNS, central nervous system; PNS, peripheral nervous system.

Other muscular functions were affected in 33/85 (39%) patients who manifested variable combinations of ptosis (22), facial diplegia (19), progressive external ophthalmoparesis (PEO) (11), mild dysphagia (10), dysarthria/dysphonia (3) and rigid spine (1) (figure 1A). Nineteen patients required gastrostomy tube because of severe dysphagia, failure to thrive or both. The nervous system was affected in 15 out of 92 (16%) causing recurrent seizures (six), encephalopathy (seven), cognitive impairment (four), sensorineural hearing loss (three) and episodic coma (one). As previously reported, abnormalities in the cerebral or cerebellar hemispheres such as atrophy (three), immature myelination (one) and lissencephaly (one) were identified on brain MRI or histological postmortem studies.^5 8^

Data on mortality are available for 79 individuals: 37 infantile, 35 childhood and 8 late onset. Because data for the late-onset group are sparse and because their survival does not differ from the childhood-onset group (P=0.84 by Wilcoxon test), we have combined these in a childhood-onset/late-onset group, which figure 1B compares with the infantile-onset group. Survival differs significantly (P<0.0001 by log-rank test). Median post-onset survival (POS) is 1.0 years (CI 0.58 to 2.33) for the infantile group and at least 23.0 years (CI 10.0 to incomputable) for the childhood-onset/late-onset group. Separate comparisons of POS of the childhood-onset and late-onset group to the infantile-onset group are provided in online supplemental figure 1A,B. Both comparisons are significant (P<0.01). Median POS is not directly estimable for the childhood group (online supplemental figure 1A). We infer that it is >13 years since that is the 40th percentile value. Median POS for the late-onset group (online supplemental figure 1B) is 23.0 years, with a very wide CI, given so few cases (0.0 to incalculable).

10.1136/jmedgenet-2017-105012.supp3 Supplementary data

Because 27.2% (25 of 92) of the patients are known to have died from respiratory failure, we also analysed time to ventilation or death (‘event-free survival’, figure 1C). The data are limited because time to ventilation use is not available for some patients. Time from onset to ventilation or death is significantly greater for the childhood-onset/late-onset group (P≤0.0001 for log-rank test). Median time to event is 1.67 (CI 0.11 to 2.33) years for infantile onset and 10.00 (CI 3.50 to 22.00) years for childhood/late onset.

### Molecular genetics and biochemical studies

Levels of mtDNA in muscle were measured in 71 patients and showed significant depletion (<30% residual mtDNA relative to age-matched control subjects) in 47 (66%). Higher proportions of infantile-onset (21/26; 81%) and childhood-onset (24/31; 77%) had mtDNA depletion than late onset (1/14; 7%) (P≤0.0001 for late-onset vs others, 1 df χ^2^ test). Multiple mtDNA deletions were detected in muscle of 24 patients primarily in late-onset patients: infantile-onset (1/8; 12.5%), childhood-onset (7/14; 50%) and late-onset (16/16; 100%). The proportions with multiple mtDNA deletions differed significantly over the three groups (P≤0.001, 2 df χ^2^ test).

**Figure 2:**
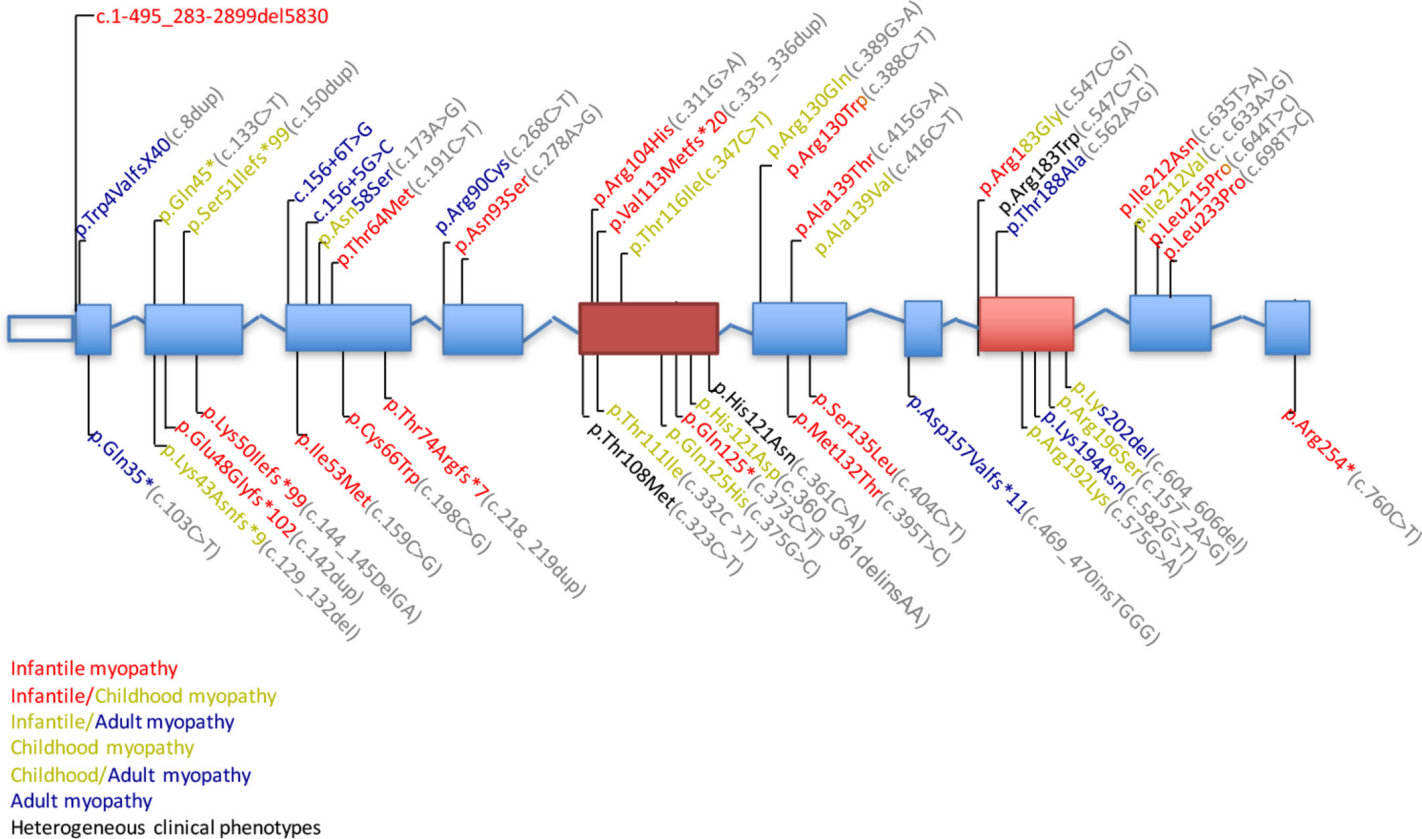
Thymine kinase 2 (*TK2*) mutations. Mutations in the coding and splice-site regions of *TK2* (NM_004614.4). Hot-spot exons are marked in red boxes. Protein changes are colour-coded based on the clinical phenotype caused in our cohort of patients. Protein changes found with more than one clinical phenotype are highlighted with multiple colours. Protein changes found in all of the three forms are noted in black. DNA sequence variants are noted in grey font.

Pathogenic variants were identified in all exons of *TK2* and no clear genotype–phenotype correlation was apparent. The most common mutation, p.Thr108Met, was identified in a homozygous state in 13 patients with heterogeneous clinical phenotypes (three infantile-onset, six childhood-onset and four late-onset individuals) (figure 2). The substrate binding site (encoded by exon 5) and active site (exon 8) are hotspots for the mutations. We identified 11 novel mutations affecting highly conserved amino acid residues, splice site regions or a reading frame (p.Lys50Ilefr99*, p.Arg104His, p.Thr116Ile, p.His121Asp, p.Met132Thr, p.Ala139Thr, c.156+6T>G, p.Asp157Valfs11*, p.His163Asp, p.Ile212Val and p.Gln125His); all were predicted to be deleterious by PolyPhen-2, SIFT or both.

TK2 activity measured in 11 patient-derived fibroblast cell lines showed residual activity ranging from 1% to 39% relative to healthy controls without correlation with clinical severity (online supplementary table 4).

10.1136/jmedgenet-2017-105012.supp2 Supplementary data

### Muscle biopsy

Muscle biopsy samples, obtained in 83 patients, were subjected to a variety of diagnostic tests. Histochemical, histological, biochemical and molecular genetics analyses had been prioritised and performed based on the differential diagnosis of mitochondrial versus other metabolic or congenital myopathies thereby limiting the opportunity to compare all tests within individual patients.

Muscle histology data were available in 36 patients. Myopathic changes included atrophic and/or necrotic fibres (28/36, 69.5%), fibre size variability/type 1 predominance (24/36, 66.6%), increased central nuclei (11/36, 30.5%), sarcoplasmic vacuoles (5/36, 13.9%), lipid droplets or fat replacement of muscle tissue (7/36, 19.5%), and fibrosis or increase of connective tissue (14/36, 38.9%). Muscle histochemistry was abnormal in all 71 patients studied: 53/71 (74.6%) had numerous cytochrome *c* oxidase (COX)–deficient and ragged-red fibres (RRFs), 14/71 (19.7%) had only COX-deficient fibres and 4/71 (5.7%) had isolated RRFs. Oxidative-phosphorylation (OXPHOS) enzyme activities were measured in muscle of 48 patients. Multiple OXPHOS defects were identified in 35/48 (73%) while single enzyme abnormalities were detected in 7/48 (14.5%) (complex IV deficiency in 4/49, complex III in 1/48, isolated increase of citrate synthase in 1/48 and complex I in 1/48). In six patients (12.5%) with childhood-onset (two) and late-onset (four) myopathies, OXPHOS activities were normal.

### Classification

Based on clinical, biochemical and molecular genetics features, we identified three clinical forms of TK2 deficiency (online supplementary tables 1–3) that we defined by the following criteria:

#### Infantile-onset myopathy

defined by onset in the first year of life (mean 0.53±SD 0.32), rapidly progressive myopathy, with or without encephalopathy and severe muscle mtDNA depletion. In our cohort of patients, 39 (42.4%) patients fulfilled criteria for TK2 infantile myopathy. Severe congenital myopathy and hypotonia or motor regression progressed rapidly to fatality in the majority of cases within 1 year of follow-up due to respiratory failure (figure 1B). Virtually all never developed the ability to walk or became wheelchair-bound by age 4 years (94%) and 89% required ventilator support. Additional clinical manifestations were seizures (seven), encephalopathy (five), lissencephaly (one), cognitive dysfunction (three), ptosis (four), facial diplegia (three), dysphagia (three) and multiple bone fractures (two). Single patients presented with each of the following clinical abnormalities: nephropathy, rigid spine, coma episodes, cardiomyopathy, bi-ventricular hypertrophy, arrhythmia, oesophageal atresia, microcephaly, bilateral optic atrophy, severe peripartum asphyxia, anaemia, thrombosis, capillary-leak syndrome, bilateral chylothorax and occipital skin necrosis. Overall, 13/39 (33.3%) infantile-onset patients had non-skeletal muscle manifestations while 10/39 (25.6%) had central nervous system (CNS) abnormalities.

#### Childhood-onset myopathy

delineated by childhood-onset (>1 and <12 years), moderately to rapidly progressive myopathy, and muscle mtDNA depletion with, in some cases, multiple deletions. All patients fulfilled criteria for childhood limb myopathy with proximal weakness, Gower signs and myopathic histological changes, but some also manifested facial diplegia (11), ptosis (9), PEO (3) and dysphagia (1). The weakness occasionally resembled spinal muscular atrophy (SMA) type 3 with progressive clinical course to wheelchair-bound status in the majority (60%) and invasive or non-invasive ventilator dependency in 55% (12/22). A minority (7/37, 18.9%) of the patients showed non-skeletal muscle involvement including hearing loss (two), as well as one each with cognitive decline, encephalopathy, prolonged QT, arrhythmia, multiple bone fractures, renal tubulopathy and gynaecomastia. Nervous system involvement was evident in 4/37 (10.8%) patients.

#### Late-onset myopathy

defined by myopathy that clearly manifests at age ≥12 years with mtDNA depletion, multiple deletions or both in muscle. Sixteen patients (17.4%) in our cohort presented with adult-onset myopathy, which mimicked facioscapulohumeral dystrophy with marked facial weakness, scapular winging or both in seven (43.75%). In retrospect, subtle signs of myopathy were often present in childhood (eg, slow running) but did not prompt medical evaluation until adulthood. All patients retained the ability to walk, but 4/9 (44.4%) used ventilator support. Additional manifestations were ptosis (nine), PEO (eight), dysphagia (six), dysarthria/dysphonia (three), respiratory insufficiency (five), cardiomyopathy (two) and one each with peripheral neuropathy and hearing loss. Non-muscle manifestations were present in 4/16 (25%), but none had CNS abnormalities.

## Discussion

A growing number of mitochondrial diseases with mtDNA instability have been linked to mitochondrial deoxynucleotide pool imbalance, including deficiencies of TK2, dGK, RRM2B or TYMP. For these disorders, treatment with nucleotides/nucleosides has ameliorated phenotypic and biochemical abnormalities in cellular and animal models.^28–31^ An understanding of the natural history of deoxynucleotide pool imbalance disorders is essential if these treatments are to be tested effectively in clinical trials. We have retrospectively documented the manifestations and natural history of TK2 deficiency in a cohort of 92 patients, including 25 recently diagnosed.

The disease presents predominantly as a myopathy with a wide spectrum of age at onset and severity.

We have confirmed the phenotypic heterogeneity of TK2 deficiency and divided the spectrum into three major clinical forms: (1) infantile-onset myopathy evident in the first year of life with rapid progression to early death; (2) childhood-onset myopathy with proximal limb weakness progressing, in most cases, to loss of ambulation within 10 years of disease onset and ventilator use; and (3) late-onset myopathy with subclinical or mild myopathy at disease onset in adolescence through adulthood and slow progression with retained ability to walk but requirement of ventilator support in nearly half. Infantile-onset and childhood-onset myopathies were the most common clinical phenotypes accounting for 82% of TK2-deficient patients. Although accounting for a small minority of the patients (18%), the late-onset form has been only recently associated with *TK2* mutations and therefore may be under-represented.

We identified three important features that enable stratification of TK2-deficient patients: (1) age at onset, (2) rate of muscle weakness progression and (3) post-onset survival. POS was dramatically shorter in the infantile-onset form compared with the childhood-onset/late-onset forms (median 1 vs 23 years). Muscle weakness was evident in the first year of life in the infantile-onset form with 26 of 32 (81%) never acquiring the ability to walk independently and virtually all (30/32; 94%) were non-ambulatory by age 4 years. In contrast, 19 of 30 (63%) of childhood-onset patients became wheelchair-bound whereas late-onset cases never became wheelchair-bound. Therefore, length of survival and progression of muscle weakness, reflected by capacity to ambulate, represents important clinical features for the evaluation of treatment efficacy in future clinical trials.

The range of weakness severity among TK2-deficient patients parallels the spectrum of SMA with infantile-onset TK2 myopathy resembling SMA type 1 and severe type 2, childhood-onset myopathy similar to milder type 2 and 3a SMA, and late-onset myopathy comparable to SMA types 3b and IV, although the weakness is more progressive.^10 28 32^ Genetic screening for TK2 deficiency must be considered in children, particularly infants, with rapidly progressing muscle weakness, especially when associated with elevated blood lactate and CK.

The adult myopathy shares prominent facial weakness with facioscapulohumeral muscular dystrophy (FSHD).^18 29^ We suggest that genetically undiagnosed patients with autosomal recessive myopathies, including FSHD-like cases, should be screened for *TK2* mutations in blood or mitochondrial alterations in muscle biopsies.

While hypotonia and proximal muscle weakness are common in all of the three clinical forms, the involvement of ocular muscles with ptosis or PEO is more frequent in late-onset cases (11/16, 68.7%) than in the childhood-onset (11/37, 29.7%) and infantile-onset forms (3/39, 7.7%). Therefore, extraocular muscle involvement is much more characteristic of TK2 late-onset form. Non-muscle manifestations noted in 27.2% of patients (25/92) were mostly CNS or peripheral nervous system involvement, cardiopathy and bone fractures. Interestingly, CNS signs are more frequent and severe in the infantile form with 10/39 patients (25.6%) manifesting as encephalopathy, seizures or cognitive impairment; however, due to the high frequency of restrictive lung disease, it is uncertain in some cases whether the cerebral manifestations might have been due to hypoxic–ischaemic damage in the setting of respiratory failure or infections. Nevertheless, these data indicate the importance of a multisystemic evaluation of patients with infantile-onset form for clinical care and interventional trials.

Skeletal muscle biopsies showed mtDNA defects ranging from severe mtDNA depletion in the infantile-onset and childhood-onset myopathies to partial mtDNA copy number reduction or multiple mtDNA deletions in isolation in the late-onset myopathy. Although a few *TK2* mutations were associated with specific clinical forms (eg, frameshift mutation in exons 2 and 3 in infantile-onset cases and p.Arg90Cys in late-onset myopathy) (figure 2), 12 mutations were associated with multiple phenotypes so overall genotype–phenotype correlations based on type of mutation and protein location could not be clearly delineated. Based on the genomic localisation of the variants, we confirmed exon 5 as a hot spot^20^ and identified exon 8 as a second frequent location for *TK2* mutations. Exon 5 encodes the α4 helix, which is required for enzyme dimerisation and nucleoside recognition in the deoxynucleoside kinase domain. Exon 8 encodes the α8 helix, an integral component of the lid region required for binding of phosphate groups of ATP and catalysis.^9 14 20^

Although progressive proximal muscle weakness was uniformly present in all three clinical forms of TK2 deficiency, the myopathy manifested variable severity and functional impairment. The majority of the patients also had respiratory muscle weakness, which was the most common cause of death. Reduced total VC was identified in both symptomatic and asymptomatic patients. In some patients with late-onset myopathy, nocturnal dyspnoea was an initial symptom of the disease.

CK levels were elevated (272–6500 U/L) in 57 TK2-deficient patients and normal in only five: one with infantile-onset myopathy and four with childhood-onset myopathy, indicating that a normal CK does not exclude TK2 deficiency. Myoglobinuria and rhabdomyolysis were rare (three patients).

EMG revealed myopathic changes in 78.6% of patients tested. Surprisingly, neurogenic changes were detected in six patients including two with sensory axonal peripheral neuropathy. One was a 58-year-old man with symptomatic sensory axonal neuropathy, but also signs of myopathy including axial and proximal limb weakness, PEO and pulmonary function tests indicating restrictive lung disease; and one an infantile patient with a severe multisystemic disorder. Other medical conditions causing peripheral neuropathy were excluded in these patients. These data demonstrate peripheral nerve involvement in a minority of patients with TK2 deficiency.

Results of routine muscle histology were available in 36 patients; in the majority of samples, myopathic changes (69.5%) and fat/connective replacement (38.9%) were noted. Histochemical data were available in 77% of patients confirming COX deficiency with or without RRFs as morphological hallmarks of TK2 deficiency. In 87.5% of the biopsies, biochemical activities of respiratory chain enzymes were reduced with isolated complex IV deficiency or variable defects of complexes I, III and IV. The combination of dystrophic features with COX defect and RRF is highly suggestive of TK2 deficiency, but must be supplemented by molecular genetic studies to confirm TK2 deficiency.

In conclusion, TK2 deficiency is a frequent mitochondrial predominantly myopathic disorder that may be under-diagnosed due to its diverse spectrum of clinical presentations. Our study provides diagnostic clinical and molecular genetic criteria and describes additional phenotypic features, as well as outcomes, that are important for early and accurate diagnosis and optimal management of this disease. Greater awareness of diagnostic criteria and the variability of clinical manifestations may lead to increased recognition of the disorder, which is particularly important as preclinical studies of pyrimidine deoxynucleoside monophosphates and deoxynucleoside therapies have demonstrated safety and efficacy in a Tk2 H126N knockin mouse model.^30 31^
