# Supplementary material for: Retrospective natural history of thymidine kinase 2 deficiency
Source: J Med Genet. 2018 Mar 30;55(8):515–21. doi: 10.1136/jmedgenet-2017-105012 (PMC6073909; doi:10.1136/jmedgenet-2017-105012)
Supplement: Supplementary data [file jmedgenet-2017-105012supp001.docx]

| **Supplementary Table 1: TK2-deficient patients with Infantile-onset myopathy** | | | | | | | | | | | | | |
| --- | --- | --- | --- | --- | --- | --- | --- | --- | --- | --- | --- | --- | --- |
| **P#** | **Age at onset/ Sex** | **W/ H Onset** | **GMF <50/ Age when WC-bound** | **Ventilator/ Gastrostomy tube** | **Other manifestations** | **mtDNA copy number**  **%** | **mtDNA**  **deletions** | **RCA** | **CK** | **EMG** | **TK2**  **mutations** | **Death/**  **Age** | **Ref.** |
| **P1** | 0.67/F | +/+ | 1.08/NW | +/+ | - | 22 | - | 🡻I, III, IV, V | 3,436 | NA | p.Ile212Asn; p.Ile212Asn | -/3 | 1 |
| **P2** | 1/F | +/+ | 1.24/NA | NA/NA | - | 22 | - | 🡻I, III, IV, V | 1,875 | NA | p.Ile212Asn; p.Ile212Asn | +/1.58 | 1 |
| **P3** | 0.67/M | +/+ | 1/NW | +/+ | - | 22 | - | 🡻I, III, IV, V | 4,000 | NA | p.Ile212Asn; p.Ile212Asn | -/3 | 1 |
| **P4** | 1/M | +/+ | 2.15/NW | +/NA | - | 14 | NA | 🡻I, II+III, I+III, IV | 1,238 | NA | p.His121Asn;  p.Thr108Met | +/3.33 | 10 |
| **P5** | 0.08/F | +/+ | 0.08/NW | +/NA | - | 14 | NA | 🡻I, II+III, I+III, IV | NA | NA | p.Ile53Met; p.Ile53Met | +/2 | 10 |
| **P6** | 1/M | +/+ | NA/NW | NA/NA | - | 20 | NA | 🡻I, III, IV, 🡹CS | 386 | NA | p.Thr108Me;t p.Thr108Met | +/3.33 | 9 |
| **P7** | 0.17/M | +/+ | NA/NW | NA/NA | Nephropathy  Encephalopathy | 60 | NA | 🡻IV | NA | NA | p.Arg183Gly; p.Arg254* | +/0.83 | 19 |
| **P8** | 0.42/F | +/+ | NA/NW | NA/NA | - | 37 | NA | 🡻I, III, IV | NA | NA | p.Glu48Glyfs*102; p.Glu48Glyfs*102 | +/1.25 | 19 |
| **P9** | 0.67/F | +/+ | NA/NW | NA/NA | - | NA | NA | NA | 1,511 | nl | p.Thr64Met;  p. Arg183Trp | +/2 | 14 |
| **P10** | 1/F | +/+ | 2/NW | NA/NA | - | 10 | NA | NA | 400 | M | p.Thr64Met;  p. Arg183Trp | +/2.33 | 14 |
| **P11** | 0.58/M | +/- | 1.08/NW | +/+ | Rigid spine  Facial diplegia | 18 | NA | NA | 1,724 | M | p.Thr108Met; p.Gln125* | +/1.58 | 12 |
| **P12** | 0.58/M | +/+ | 5/NW | NA/NA | Cognitive impairment  Seizure  Coma episodes | NA | NA | NA | nl | NA | p.Cys66Trp; p.Leu215Pro | +/7 | 5 |
| **P13** | 0.5/M | +/+ | 4/4 | NA/NA | Encephalopathy  Seizure  Ptosis  Cognitive impairment | 8.40 | NA | NA | NA | M | p.Cys66Trp; p.Leu215Pro | +/6 | 5 |
| **P14** | 0.67/F | +/+ | NA/NA | NA/NA | - | NA | NA | 🡻I, III, IV | 🡹 | NA | NA  (sister of patient P51) | +/1.16 | 4 |
| **P15** | 0.58/F | +/+ | 1.32/NW | +/+ | Seizure  Encephalopathy Cardiomyopathy  Fractures of large bones | 10 | NA | 🡻I+III, II+III, IV, II, 🡹 CS | 1,270 | M | p.Arg130Trp;  p.Arg183Trp | +/10 | 6 |
| **P16** | 0.42/F | +/+ | 0.5/NA | NA/+ | NA | NA | NA | NA | 3,670 | M | p.Arg130Trp;  p.Arg183Trp | +/1 | 6 |
| **P17** | 0.16/F | +/+ | NA/NW | NA/+ | Seizure | 10 | NA | NA | 3,015 | M | p.Arg130Trp; p.Arg130Trp | +/0.58 | 6 |
| **P18** | 0.25/F | +/+ | 0.41/NW | NA/+ | Seizure | NA | NA | NA | 1,996 | M | p.Arg130Trp; p.Arg130Trp | +/0.58 | 6 |
| **P19** | 0.25/M | +/+ | NA/NW | NA/NA | - | NA | NA | NA | NA | M | p.Arg130Trp; p.Arg130Trp | +/0.5 | 6 |
| **P20** | 0.25/M | +/+ | 0.41/NW | NA/+ | - | NA | NA | NA | 520 | M | p.Arg130Trp; p.Arg130Trp | +/0.66 | 6 |
| **P21** | 0.05/F | +/- | NA/NW | +/NA | Seizure  Encephalopathy | 5 | NA | 🡻I, III, IV | 1,705 | M | p.Arg130Trp;  p.Thr74Argfs*7 | +/0.25 | 8 |
| **P22** | 0.25/M | +/+ | 0.66/NW | NA/NA | NA | NA | NA | NA | NA | NA | p. Ile212Asn;  c.1-495_283-2899del5830 | +/0.66 | 20 |
| **P23** | 0.25/M | +/+ | 0.66/NW | NA/NA | NA | 20 | NA | NA | 3,600 | NA | p. Ile212Asn;  c.1-495_283-2899del5830 | +/0.66 | 20 |
| **P24** | 0.25/M | +/+ | 0.5/NW | NA/NA | NA | NA | NA | NA | NA | NA | p. Ile212Asn;  c.1-495_283-2899del5830 | +/0.5 | 20 |
| **P25** | 0.42/M | +/+ | NA/NW | +/NA | Bi-ventricular hypertrophy | NA | NA | NA | 820 | NA | p. Arg183Trp;  p.Ser135Leu | -/1.83 | 20 |
| **P26** | 0.75/F | +/+ | 0.83/NW | NA/NA | NA | 22 | NA | 🡻I, II+III, IV | 3,000 | NA | p.Ser135Leu; p.Asn58Ser | -/1.20 | 20 |
| **P27** | 0.75/M | +/+ | 3/NA | +/- | Ptosis | 25 | NA | 🡻I, I+III,II+III,IV | 2,500 |  | p. Arg183Trp; p. Arg183Trp | -/4 | 20 |
| **P28** | 0.50/M |  | NA/1.5 | -/- | NA | 5 | - | 🡻I, I+III, II+III, IV 🡹CS | 400-1,600 | NA | p. Arg183Trp; p.Ala139Thr | +/3 | UP |
| **P29** | 0.92/F | +/+ | -/4 | -/- | Facial diplegia | NA | NA | NA | 554 | M | p.His121Asn;  p.Thr108Met | -/4 | UP |
| **P30** | 0.92/M | +/- | NA/NA | +/NA | NA | 31 | - | 🡻II+III, IV | NA | NA | p.Arg104His; p.Arg130Trp | NA | UP |
| **P31** | 0.75/F | +/+ | NA/NA | NA/NA | NA | 39 | - | NA | 783-3,500 | NA | p.Thr108Met; p.Thr108Met | NA | UP |
| **P32** | 0.50/F | +/+ | NA/NA | NA/NA | Abnormal LFTs  Dysphagia | 29 | - | NA | 1,434-2,197 | NA | p.Arg130Trp; p.Arg130Trp | NA | UP |
| **P33** | 0.83/M | +/+ | NA/NA | +/- | Cognitive impairment  Dysphagia | 10 | NA | 🡻I, IV | 2,000 | NA | p.Gln125*;  p.Met132Thr | +/1.5 | UP |
| **P34** | 0.00/F | +/+ | 44/44 | +/- | Ptosis | 30 | + | 🡻I, III, IV | nl | M | p.Thr108Met; p.Thr108Met | -, 44 | 3 |
| **P35** | 1/M | +/+ | 1.58/NW | +/+ | - | NA | NA | NA | NA | NA | p.Lys50Ilefr*99;  p.Thr108Met | -/ 2 | UP |
| **P36** | 1/M | +/+ | 1/NA | +/+ | Esophageal atresia | 14 | NA | NA | 4,700 | M | p.Arg130Trp;  p. Arg183Trp | +/1.5 | UP |
| **P37** | 0/M | +/+ | 0/NW | +/+ | lissencephaly/pachy- gyria, microecephaly, bilateral optic atrophy, severe peripartal asphyxia, respiratory insufficiency, anemia, thrombosis, capillary- leak syndrome, bilateral chylothorax, and occipital skin necrosis. | 1 | NA | NA | NA | NA | c.−270+2561del-ins; 7287–7335inv  c.−270+2561del-ins; 7287–7335inv | +/0.15 | 23 |
| **P38** | 0.41/M | +/+ | 0.6/NW | +/+ | NA | NA | NA | NA | NA | NA | p.Met117Val;  p.Ala139Val | +/1 | 24 |
| **P39** | 0.6/M | +/+ | 0.6/NW | +/+ | Epilepsy, brain atrophy, axonal peripheral neuropathy, multiple fractures, ptosis, dysphagia, facial diplegia | 20 | NA | NA | 3000-6500 | N | p.Met117Val;  p.Ala139Val | +/12 | 24 |

| **Supplementary Table 2: TK2-deficient patients with childhood-onset myopathy** | | | | | | | | | | | | | |
| --- | --- | --- | --- | --- | --- | --- | --- | --- | --- | --- | --- | --- | --- |
| **P#** | **Age at onset/ Sex** | **W/ H Onset** | **GMF <50/ Age when WC-bound** | **Ventilator/ Gastrostomy tube** | **Other manifestations** | **mtDNA copy number**  **%** | **mtDNA**  **deletions** | **RCA** | **CK** | **EMG** | ***TK2 and* TK2**  **mutations** | **Death/**  **Age** | **Ref.** |
| **P40** | 2/F | +/+ | 3/3 | NA/NA | - | 22 | - | 🡻I, III, IV, V | 908 | NA | p.His121Asn; p.His121Asn | +/4 | 1 |
| **P41** | 1.33/F | +/+ | 4/2 | -/NA | - | 14 | NA | 🡻I, II+III, I+III, IV | 950 | NA | p.His121Asn;  p.Thr108Met | -/4 | 10 |
| **P42** | 1.25/M | +/+ | 2/2 | -/NA | - | 14 | NA | 🡻I, III, IV | 🡹 | NA | p.Ile53Met; p.Ile53Met | -/4 | 10 |
| **P43** | 2.33/M | +/- | NA | -/- | - | 8 | - | 🡻I, III, IV | 🡹 | M | p.Thr108Met;  p.Arg192Lys | -/12 | 17 |
| **P44** | 2/M | +/+ | 3/3 | +/+ | Ptosis | NA | NA | 🡻II+III, IV  🡹 CS, SDH | 577 | M/N | p.Val113Metfs20*; p.Ile212Asn | +/16 | 12 |
| **P45** | 2/F | +/+ | NA | -/- | Facial diplegia | 21 | NA | IV | 823 | N | p.Val113Metfs20*; p.Asn93Ser | -/9 | 12 |
| **P46** | 2/F | +/+ | NA/NA | NA/NA | - | 25 | NA | 🡻I, II+III,IV  🡹 CS | 790 | M | p.His121Asn;  p.His121Asn | +/6 | 12 |
| **P47** | 2/M | +/+ | 5/5 | NA/NA | Cognitive impairment  Ptosis Ophthalmoparesis | 5.7 | NA | NA | 1,850 | NA | p.Ala139Val; p.Ala139Val | -/5 | 5 |
| **P48** | 2/F | +/+ | 2.5/NA | NA/NA | NA | 4.3 | NA | 🡻I, III, IV | 2,200 | NA | p.Ala139Val; p.Ala139Val | -/2.49 | 5 |
| **P49** | 3/M | +/+ | NA/NA | NA/NA | Encephalopathy  Ptosis Ophthalmoparesis | 9.2 | NA | NA | 523 | M | p.Cys66Trp; p.Leu215Pro | -/8 | 5 |
| **P50** | 2.5/M | +/- | 9/9 | NA/NA | Restrictive respiratory insufficiency | 100 | - | 🡻I , II+III,I+III 🡹 II, IV | nl | NA | p.His121Asn; p.His121Asn | -/9 | 7 |
| **P51** | 1.08/M | +/+ | 1.41/NW | NA/NA | - | 5.0 | NA | 🡻I, IV | 802 | NA | p.Gln45*; p.Asn58Ser | +/1.5 | 4 |
| **P52** | 1.5/F | +/+ | 2/2.25 | NA/+ | - | 5.0 | NA | 🡻I+III, II+III, IV, II  🡹CS | 1315 | M/N | p.Arg183Trp; p.Arg183Trp | +/3 | 6 |
| **P53** | 1.25/M | +/+ | 3/3 | +/+ | Ptosis  Facial diplegia Prolonged QT Incomplete right bundle branch block | 24 | NA | 🡻IV | 570 | NA | p.Lys43Asnfs*9; p.Arg130Gln | -,/15 | 21 |
| **P54** | 2.5/M | +/- | 5/NA | +/- | Hearing loss | 10 | NA | 🡻I, III, IV | 540 | M | p.Ser51Ilefs*99;  p.Lys202del | +/8.5 | 11 |
| **P55** | 4/M | +/- | NA/NA | NA/NA | NA | NA | NA | NA | 2,155 | NA | p.Ile53Met;  p.Arg196Ser | -/10 | 20 |
| **P56** | 1.16/M | +/+ | NA/NA | +/NA | NA | 5 | NA | 🡻III,IV | 773 | NA | p.Thr108Met p.Leu233Pro | +/3 | 20 |
| **P57** | 1.5/M | +/+ | 2.66/1.83 | NA/NA | NA | 15 | NA | NA | 764.7 | NA | c.156+5G>C;  p.Thr111Ile | +/3.5 | 22 |
| **P58** | 3/M | NA | 16/NA | -/- | NA | 10 | - | 🡻I, I+III, II+III, IV  🡹 CS | 703 | NA | p.Thr108Met; p.Thr108Met | -/16 | UP |
| **P59** | 1.5/M | +/+ | NA/NA | +/- | PEO,  Facial diplegia  Multiple bone fractures  Myoglobinuria | NA | NA | NA | 1000 | NA | p.His121Asn; p.His121Asn | -/25 | 20 |
| **P60** | 2/M | +/+ | 8/8 | -/- | Ptosis | 10 | NA | 🡻I, III, IV, V | 425 | nl | p.Lys202del; p.Arg183Gly | -/8 | UP |
| **P61** | 1.17/M | +/+ | 1.17/1.17 | +/- | Neck weakness | 17 | - | 🡻I, III, IV | 5,500 | M | p.His121Asp; p.Arg192Lys | -/ 3 | UP |
| **P62** | 1.5/M | +/+ | 2/2 | +/+ | Mild facial diplegia  Dysphagia  Sensorineural hearing loss | 15 | - | 🡻I, III, IV | 274 | M | p.Arg130Trp; p.Ile212Val | -/ 3 | UP |
| **P63** | 11/F | +/- | 19/NA | -/- | Facial weakness | NA | NA | NA | nl | NA | p.Thr116Ile; p.Thr116Ile | -/ 19 | UP |
| **P64** | 4.42/M | +/- | 8/NA | NA/NA | - | 24 | + | NA | 260-1,100 | NA | p.Ser51fs;  p.Gln125His | -/8 | UP |
| **P65** | 3.17/M | NA | NA/NA | NA/NA | Renal tubulopathy | 12 | - | 🡻IV | NA | NA | p.Asn58Ser; p.Asn58Ser | NA | UP |
| **P66** | 1.17/M | +/+ | NA/2 | NA/NA | - | 20 | NA | NA | 905 | NA | p.Thr108Met; p.Thr108Met | +/3 | 9 |
| **P67** | 1.33/F | +/+ | 1.91/2 | NA/NA | - | 20 | NA | NA | nl | NA | p.Thr108Met; p.Thr108Met | +/1.91 | 9 |
| **P68** | 4/M | +/- | 15/15 | -/- | Facial weakness, Scapular winging  Severe Achilles tendon contractures | 35 | + | NA | nl | M | p.Arg130Trp; p.Lys202del | -/15 | UP |
| **P69** | 9/M | +/- | NA/NA | +/- | Ptosis, facial weakness, Severe Achilles tendon contractures | 50 | + | NA | 363 | M | p.His121Asn; p.His121Asn | -/9 | UP |
| **P70** | 3/M | +/+ | 13/13 | +/NA | Ptosis  Facial weakness | 100 | - | nl | NA | NA | p.Arg183Gly; p.Lys202del | -/25 | 16 |
| **P71** | 9/F | +/- | 28/NA | +/- | - | NA | NA | nl | NA | NA | p.Asn58Ser; p.Asn58Ser | -/28 | 20 |
| **P72** | 2/M | +/- | 20/22 | +/- | Facial weakness  Scapular winging  Gynecomastia | 45 | + | 🡻I  🡹CS | 757 | M | p.Thr108Met; p.Thr108Met | -/22 | 13 |
| **P73** | 4/F | +/- | 15/15 | +/+ | Ptosis, facial weakness, scapulohumeral weakness, neck flexion weakness | 31 | + | 🡻I, III, IV | 551 | M | p.Thr116Ile; p.Thr116Ile | -/18 | UP |
| **P74** | 1.1/F | +/- | NA/NA | -/- | Ptosis  Facial diplegia | 17 | + | 🡻I, III, IV | 2,435 | NA | p.Thr108Met; p.Thr108Met | -/29 | UP |
| **P75** | 4/M | +/NA | NA/NA | NA | Pelvic and scapular limb-girdle atrophy | 57 | + | nl | NA | M/N | p.Thr108Met; p.Thr108Met | NA | 25 |
| **P76** | 1.1/F | +/- | NA/NA | -/NA | Neck weakness | NA | NA | NA | 1560 | M | p. Arg183Trp;  p. Arg183Trp | +/1.91 | UP |

| **Supplementary Table 3: TK2 deficient patients with late-onset myopathy** | | | | | | | | | | | | | |
| --- | --- | --- | --- | --- | --- | --- | --- | --- | --- | --- | --- | --- | --- |
| **P#** | **Age at onset/ Sex** | **W/ H Onset** | **GMF <50/ Age when WC-bound** | **Ventilator/ Gastrostomy tube** | **Other manifestations** | **mtDNA copy number**  **%** | **mtDNA**  **deletions** | **RCA** | **CK** | **EMG** | **TK2**  **mutations** | **Death/**  **Age** | **Ref.** |
| **P77** | NA/M | +/+ | - | -/- | Moderate restrictive respiratory insufficiency  Facial weakness | 30 | + | nl | 272 | M | p.Thr108Met; p.Thr108Met | -/31 | 3 |
| **P78** | 20/F | +/- | - | +/+ | Facial diplegia | 20 | + | 🡻I, III, IV | 1,400 | M | p.Thr108Met; p.Thr108Met | +/43 | UP |
| **P79** | 13/M | +/+ | - | -/- | Mild restrictive respiratory insufficiency | 30 | + | NA | 647 | M | p.Trp4ValfsX40;  p.Arg90Cys | -/31 | 3 |
| **P80** | NA/M | +/NA | NA/NA | NA | Rhabdomyolysis  Congenital cardiomyopathy | 166 | + | nl | NA | NA | p.Lys202del;  p.Thr108Met | NA | 25 |
| **P81** | 30/F | +/NA | NA/NA | NA | Ptosis  Dysphonia  Dysphagia  Limb-girdle weakness | 61 | + | 🡻III | NA | NA | p.Lys202del; p.Lys202del | NA | 25 |
| **P82** | 36/F | NA | NA/NA | NA | Ptosis  Facial diplegia | 80 | + | 🡻I, 🡹CS | NA | NA | p.Lys202del;  p.Lys202del | NA | 25 |
| **P83** | 50/M | +/NA | NA/NA | NA | Limb-girdle weakness Dysphagia, Dysarthria, PEO, facial diplegia | 183 | + | NA | NA | NA | p.Lys202del;  p.Lys202del | NA | 25 |
| **P84** | 21/F | +/NA | NA/NA | NA | Dysphagia, Respiratory insufficiency | 39 | + | 🡹CS | NA | nl | p.Thr108Met; p.Thr108Met | NA | 25 |
| **P85** | 13/M | +/NA | NA/NA | NA | Rhabdomyolysis  Hypertrophic cardiomyopathy, ptosis | 42 | + | NA | 🡹 | M | p.Arg192Lys; p.Thr108Met | NA | 25 |
| **P86** | 40/F | +/- | - | -/- | PEO, ptosis | 60 | + | NA | NA | M | p.Arg183Trp; p.Thr188Ala | +/50 | 15 |
| **P87** | 40/F | +/- | - | -/- | PEO, ptosis | NA | + | NA | 🡹 | M | p.Arg183Trp; p.Thr188Ala | +/50 | 15 |
| **P88** | 72/F | +/- | - | +/- | PEO, ptosis  Facial weakness, Sensorineural hearing loss | 183 | + | NA | NA | NA | p.Gln35*;  p.Lys194Asn | -/82 | 18 |
| **P89** | 46/F | +/- | - | -/- | PEO, ptosis  Dysphagia,  Facial diplegia Neck weakness | 50 | + | NA | 537 | M | p.Asp157Valfs*11;  c.156+6T>G | -/47 | UP |
| **P90** | 35/NA | +/- | - | +/- | PEO, ptosis  Dysphagia  Mild restrictive respiratory insufficiency  Sensory axonal neuropathy | NA | + | nl | 357 | N | p.Lys202del;  p.Lys202del | -/58 | UP |
| **P91** | 60/M | +/- | NA/NA | NA | PEO, ptosis  Respiratory Insufficiency | 43 | + | NA | NA | NA | p.Ala139Thr;  p.Arg183Trp | +/NA | UP |
| **P92** | 50/M | +/- | NA/NA | +/- | PEO  Dysarthria, Dysphagia | 100 | + | NA | NA | M | p.Thr108Met; p.Thr108Met | -/50 | UP |

P# = patient number; NA = not available; NW = never walked; - = absent; + = present; nl = normal; GMF =Global Motor Function; WC-bound = wheelchair bound (year); Death/Age=+(died), -(alive), age at last follow-up (year); W = weakness; H = hypotonia; RCA = respiratory chain activities (muscle); I = NADH dehydrogenase; II = succinate dehydrogenase; III = CoQH_2_-cytochrome *c* reductase; II+III = Succinate-cytochrome *c* reductase; I+III = NADH cytochrome *c* reductase; IV = cytochrome *c* oxidase; V = ATP synthase; CS = citrate synthase; PEO = progressive external ophthalmoplegia; M = myopathic; N = neuropathic; and UP = unpublished
