# Supplementary figures and images for: Retrospective natural history of thymidine kinase 2 deficiency
Source: J Med Genet. 2018 Mar 30;55(8):515–21. doi: 10.1136/jmedgenet-2017-105012 (PMC6073909; doi:10.1136/jmedgenet-2017-105012)

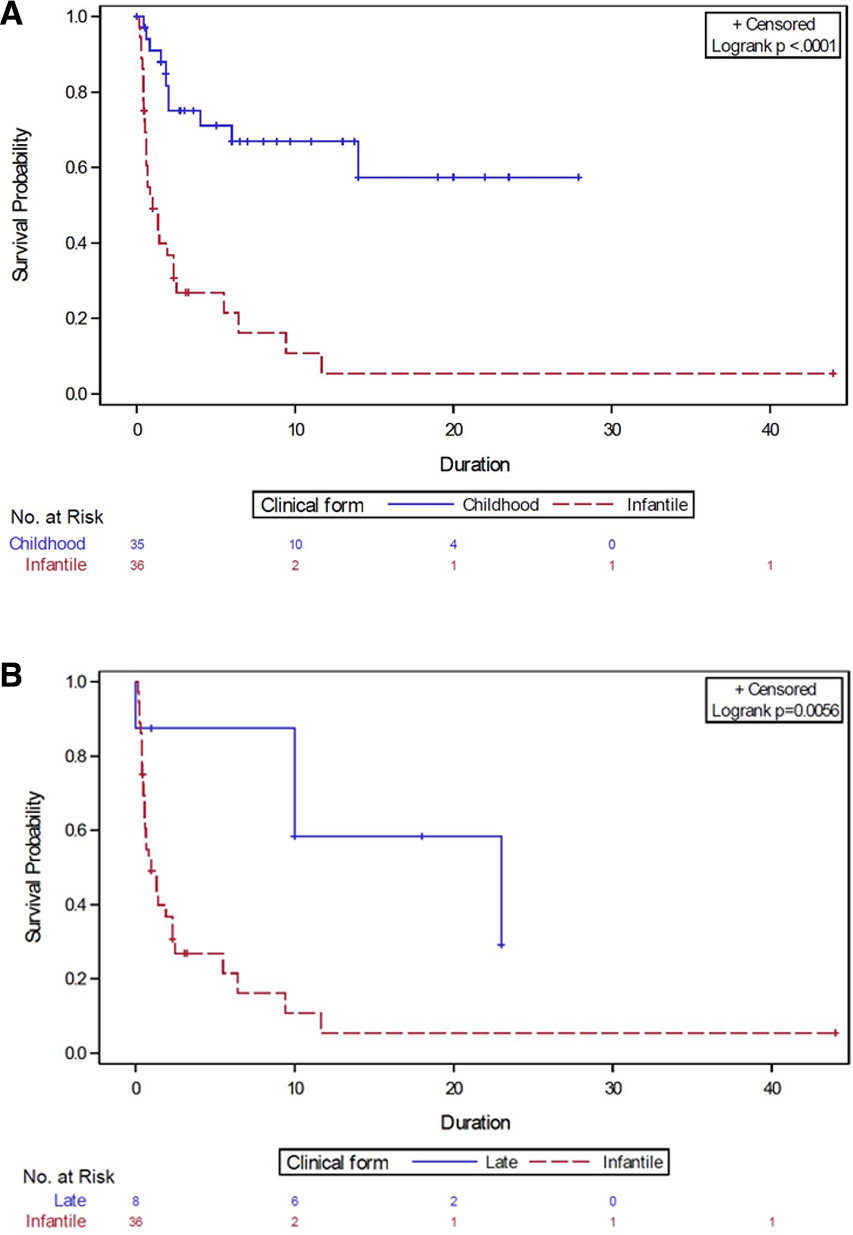

Supplement: Supplementary data [file jmedgenet-2017-105012supp003.jpg]
