# Supplementary material for: Retrospective natural history of thymidine kinase 2 deficiency
Source: J Med Genet. 2018 Mar 30;55(8):515–21. doi: 10.1136/jmedgenet-2017-105012 (PMC6073909; doi:10.1136/jmedgenet-2017-105012)
Supplement: Supplementary data [file jmedgenet-2017-105012supp002.docx]

| **Clinical form** | **TK2 Mutation** | **Age at Death (years)** | **TK2 activity in Fibroblasts (percent of normal mean)** | **References** |
| --- | --- | --- | --- | --- |
| Adult myopathy | p.Arg183Gly; p.Lys202del | - | 1% | 16 |
| Childhood | p.Ala139Val; p.Ala139Val | - | 25.9% | 5 |
| Childhood | p.Ala139Val; p.Ala139Val | - | 14.8% | 5 |
| Childhood | p.Cys66Trp; p.Leu215Pro | **-** | 19.7% | 5 |
| Infantile | p.Arg130Trp; p.Thr74Argfs*7 | 0.25 | 4% | 8 |
| Childhood | p.Ser51Ilefs*99; p.Lys202del | 8 | 6% | 11, 25 |
| Adult myopathy | p. Arg183Trp; p.Thr188Ala | 50 | 13% | 15 |
| Adult myopathy | p. Arg183Trp; p.Thr188Ala | 50 | 25% | 15 |
| Adult myopathy | p. Lys202del; p. Lys202del | NA | 3% | 25 |
| Adult myopathy | p.Lys202del; p. Lys202del | NA | 6% | 25 |
| Childhood | p.His121Asp; p.Arg192Lys | - | 39% | Patient 1 |
| Childhood | p.Arg130Trp; p.Arg130Trp | - | 16% | Patient 2 |
| Childhood | p.Arg130Trp; p.Lys202del | - | 10% | Patient 3 |
